# The Role of lncRNAs in the Distant Metastasis of Breast Cancer

**DOI:** 10.3389/fonc.2019.00407

**Published:** 2019-05-31

**Authors:** Yinan Wu, Anwen Shao, Liangliang Wang, Kaimin Hu, Chengcheng Yu, Chi Pan, Suzhan Zhang

**Affiliations:** ^1^Cancer Institute (Key Laboratory of Cancer Prevention and Intervention, China National Ministry of Education), School of Medicine, The Second Affiliated Hospital, Zhejiang University, Hangzhou, China; ^2^Department of Neurosurgery, School of Medicine, Second Affiliated Hospital, Zhejiang University, Hangzhou, China; ^3^Interdisciplinary Institute of Neuroscience and Technology, Qiushi Academy for Advanced Studies, Zhejiang University, Hangzhou, China; ^4^Department of Surgical Oncology, School of Medicine, The Second Affiliated Hospital, Zhejiang University, Hangzhou, China; ^5^Department of Orthopedics, School of Medicine, The Second Affiliated Hospital, Zhejiang University, Hangzhou, China

**Keywords:** long non-coding RNA, breast cancer, metastasis, invasion, mechanism

## Abstract

Breast cancer (BC) remains the most frequently diagnosed cancer worldwide. Among breast cancer patients, distant metastasis and invasion is the leading cause of BC related death. Recently, long non-coding RNAs (lncRNAs), which used to be considered a genetic byproduct (owing to their unknown biological function), have been reported to be highly implicated in the development and progression of BC. In this review, we produce a summary of the functions and mechanisms of lncRNAs implicated in the different distant metastases of BC. The functions of lncRNAs have been divided into two types: oncogenic type and tumor suppressor. Furthermore, the majority of them exert their roles through the regulation of invasion, migration, epithelial—mesenchymal transition (EMT), and the metastasis process. In the final part, we briefly addressed future research prospects of lncRNAs, especially the testing methods through which to detect lncRNAs in the clinical work, and introduced several different tools with which to detect lncRNAs more conveniently. Although lncRNA research is still in the initial stages, it is a promising prognosticator and a novel therapeutic target for BC metastasis, which requires more research in the future.

## Introduction

In past years, breast cancer (BC) has always been the most frequently diagnosed cancer worldwide, and is also the major reason for cancer-related mortality in women. It is estimated that there will be 266,120 new cases of female breast cancer and an estimated 40,920 people will die of this disease in the United States in 2018 (https://www.cancer.gov/types/common-cancers). Among these patients, distant metastatic invasion has been proposed as the leading cause of death (1–4). According to an estimation, 20–30% of patients with breast cancer will develop metastasis following diagnosis (5).

Long non-coding RNAs (lncRNAs), which are longer than 200 nucleotides in length, are one subtype of RNA transcripts which used to be considered a genetic byproduct (owing to their unknown biological function) (6). In recent years, the human genome has been gradually transcribed based on the development of DNA sequencing technologies, and it is unbelievable that more than 98% of genes of the whole human genome are non-encoding, receiving a considerable amount of concern in recent years. Thanks to biological techniques and high-throughput sequencing, such as SHAPE probing (7), ChIRP (8), and CHART (9), which can be used to reveal RNA structures and RNA–RNA, RNA–DNA, and RNA–protein interactions, researchers have uncovered a great amount of lncRNAs in past years, which have played significant roles in various biological processes, especially in the progression of malignant tumors (10, 11). LncRNAs can be classified into five categories—(1) sense, (2) antisense, (3) bidirectional, (4) intronic, or (5) intergenic—with respect to the nearest protein-coding transcripts (12). A diversity of malignant tumors has been reported to be involved with lncRNAs. In these studies, lncRNAs seem to play an indispensable role in the progression of cancer, both in the development of the primary tumor and in the metastatic procedure (13, 14).

Numerous lncRNAs have been reported to be highly implicated in the development of BC (15–18), which could be categorized into two types: oncogenic type and tumor suppressor. Regardless of whether they act as a promoter or inhibitor during the progression of BC, the mechanism generally covers the following aspects: influence proliferation, invasion, apoptosis and drug resistance of BC cells. However, the molecular mechanisms in respect of how lncRNAs come into play in the distant metastasis of breast cancer remain insufficient. The functions and mechanisms of these lncRNAs—such as MALAT1 (19), HOTAIR (20) and NEAT1 (21)—have already been widely accepted regarding their interaction in the metastasis of BC. On account of the significant role that lncRNAs have played in BC metastasis, it is an urgent requirement to explore them as predictors for prognosis and as novel targets for therapy in BC patients.

With the inactivation of embryo dedifferentiation, epithelial–mesenchymal transition (EMT) increases the movement and proliferation of cancer cells (22). Transient EMT is thought to be the first necessary step in metastatic processes (23), which contributes to the migration and invasion of cancer cells, so that cancer cells leave the primary focus and form distant metastasis (24). In recent years, some researchers have indicated that EMT plays an indispensable role in the potential mechanism leading to the distant metastasis of BC (25).

**Figure 1:**
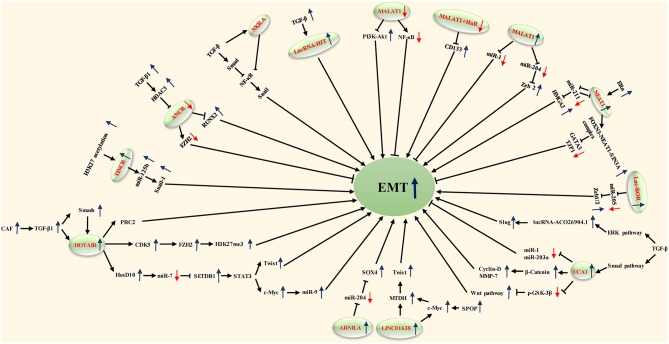
LncRNAs that are known to regulate EMT processes and their validated targets.

In this review, we produce a summary of the functions and mechanisms of lncRNAs implicated in the different distant metastases of BC (Table 1), especially via the EMT program (Table 2 and Figure 1).

**Table 1:** LncRNAs implicated in invasion, migration, EMT and metastasis of BC.

**LncRNA**	**Description**	**Classification**	**Metastasis**	**Injection place**	**Function**	**Functions in BC**	**Potential mechanism**	**Ref**.
MALAT1	Metastasis-associated lung adenocarcinoma transcript 1	Intergenic	Lung	Tail vein	Tumor suppressor	↓Invasion, migration, metastasis	MALAT1 suppresses metastasis in a TEAD-dependent manner, which associates and inhibits the prometastatic transcription factor TEAD through binding to its target gene promoters and co-activator YAP.	(26)
MALAT1	Metastasis-associated lung adenocarcinoma transcript 1	Intergenic	Lung	Coupled abdominal mammary glands	Oncogenic	↑Invasion, migration, metastasis, EMT	Knocking down of MALAT1 in the 4T1 cells, lung metastasis and inflammatory responses were significantly reversed. TNF-α level in the supernatants was decreased sharply, accompanied by the weakened ability of invasion and migration induced by LPS.	(27)
HOTAIR	Homeobox transcript antisense RNA	Antisense	Lung	Tail vein	Oncogenic	↑Invasion, metastasis	HOTAIR expression in epithelial carcinoma cells led to genome-wide re-targeting of PRC2 to an occupancy pattern more like embryonic fibroblasts, resulting in gene expression, increased cancer metastasis as well as invasiveness depend on PRC2.	(20)
HOTAIR	Homeobox transcript antisense RNA	Antisense	Lung	Mammary fat pads	Oncogenic	↑Invasion, migration, metastasis, EMT	CAFs promoted the metastatic activity of breast cancer cells by activating the transcription of HOTAIR via TGF-β1 secretion	(28)
NEAT1	Nuclear enrich abundant transcript 1	Intergenic	Lung	Left abdominal mammary fat pad	Oncogenic	↑Invasion, dissemination, metastasis, EMT	NEAT1 acts as a pivotal part in BC metastasis via the ERa-NEAT1-FOXN3/NEAT1/SIN3A-GATA3 axis.	(29)
NEAT1	Nuclear enrich abundant transcript 1	Intergenic	Lung	Tail vein	Oncogenic	↑Invasion, EMT, metastasis,	LncRNA NEAT1 induced EMT through the miR-211/HMGA2 axis.	(21)
Linc-ROR	Long Non-Coding RNA Reprogramming	Intergenic	Lung	Lateral tail veins	Oncogenic	↑Invasion, migration, metastasis, EMT	Linc-ROR functions as an important regulator of EMT and can promote breast cancer progression and metastasis through regulation of mi-205.	(30)
UCA1	Urothelial carcinoma-associated 1	Intergenic	Lung	Mammary fat pads	Oncogenic	↑Invasion, migration, metastasis, EMT	AC026904.1 and UCA1 could cooperatively upregulate Slug expression at both transcriptional and post-transcriptional levels, exerting key roles in TGF-β-induced EMT.	(31)
TINCR	Terminal differentiation-induced non-coding RNA	Intronic	Lung	Lateral tail vein	Oncogenic	↑Invasion, migration, metastasis, EMT	TINCR located in the cytoplasm of BC cells and have the ability to sponge miR-125b, upregulating the expression of miR-125b-targeted Snail-1 could reverse inhibited invasion, EMT, and migration resulted from silencing of TINCR.	(32)
BORG	BMP/OP-Responsive Gene	-	Lung	Lateral tail vein	Oncogenic	↑Invasion, migration, metastasis, EMT	BORG induces the metastatic colonies of potent BC cells by activating the transcriptional repressive activity and localization of TRIM28, which combines with BORG and leads to a great amount of changes in cancer progression.	(33)
LincIN	A long intergenic non-coding RNA between ITGB1 and NRP1	Intergenic	Lung	Tail vein	Oncogenic	↑Invasion, migration, metastasis,	LincIN exerts a critical role in translational alterations by regulating p21 as well as interacting with NF90, consequently leads to invasiveness and metastasis of BC cells.	(34)
ANCR	Anti-differentiation ncRNA	Intergenic	Lung	Tail vein	Tumor suppressor	↓Invasion, migration, metastasis, EMT	Linking ANCR interaction with EZH2 to promote its phosphorylation that facilitates EZH2 degradation and suppresses breast cancer progression.	(35)
Lnc015192	-	-	Lung	Tail vein	Oncogenic	↑Invasion, migration, metastasis, EMT	Lnc015192 and Adam12 all have the function to promote metastasis of BC and maybe partly through sponging miR-34a via the ceRNA mechanism.	(36)
LINC01638	Long intergenic non-protein coding RNA 1683	Intergenic	Lung	Tail vein xenograft	Oncogenic	↑Invasion, metastasis, EMT	LINC01638 interacts with c-Myc to prevent SPOP-mediated c-Myc ubiquitination and degradation. C-Myc transcriptionally enhances MTDH (metadherin) expression and subsequently activates Twist1 expression to induce EMT.	(37)
NKILA	NF-KappaB Interacting lncRNA	-	Lung and liver	Tail vein	Tumor suppressor	↑Apoptosis, ↓invasion	NKILA Inhibits NF-kB-mediated breast cancer metastasis	(38)
NKILA	NF-KappaB interacting lncRNA	-	Lung and liver	Mammary fat pads	Tumor suppressor	↓Invasion, migration, metastasis, EMT	TGF-β activates the NF-κB pathway. Inhibition of NF-κB signaling markedly abrogates TGF-β-induced EMT, the NKILA-mediated negative feedback affects TGF-β-induced NF-κB activation.	(39)
ARNILA	AR negatively induced lncRNA	-	Lung and liver	Tail vein	Oncogenic	↑Invasion, migration, metastasis, EMT	ARNILA functioned as a ceRNA for miR-204 to facilitate expression of its target gene Sox4, thereby promoting EMT, invasion and metastasis of TNBC.	(40)
MALAT1	Metastasis-associated lung adenocarcinoma transcript 1	Intergenic	Lung and liver	An orthotopic injection	Oncogenic	↑Invasion, migration, metastasis	MiR-1 inhibits metastasis of BC cells by targeting MALAT1	(19)
Lnc-BM	LncRNA associated with BCBM.RP11-355I22.7 (AK055647)	-	Brain	Intracardiac injection or intra-arterial injections	Oncogenic	↑Invasion, migration, vascular co-option	Lnc-BM drove STAT3-dependent expression of CCL2and ICAM1, which acts as a mediator in the process of recruitment of macrophages and vascular co-option in the cerebrum. Macrophage which have been recruited inversely produced IL-6 and oncostatin M, thereby further activating the Lnc-BM/JAK2/STAT3 pathway and promoting BM.	(41)
XIST	X-inactive– specific transcript	Intergenic	Brain and lung	Left cardiac ventricle	Tumor suppressor	↓Invasion, migration, metastasis, EMT	Decreased expression of XIST stimulated EMT and activated c-Met via MSN-mediated protein stabilization, which resulted in the promotion of stemness in the tumor cells.	(42)
HOTAIR	Homeobox transcript antisense RNA	Antisense	Lungs, kidneys and adrenalin glands	Tail vein	Tumor suppressor	↓Invasion, migration, metastasis, EMT	MiR-7 inhibits EMT and metastasis through downregulation of the STAT3 pathway. MiR-7 expression is suppressed by HOTAIR.	(43)

*LncRNA, long non-coding RNA; EMT, epithelial-mesenchymal transition; BC, breast cancer; Ref, reference*.

**Table 2:** LncRNAs implicated in the EMT program of BC.

**lncRNA**	**Publication time**	**EMT markers**	**Potential mechanism**	**References**
MALAT1	2016	↑N-cadherin	The failure to form or stabilize a repressive complex consisted of MALAT1 and HuR upregulates CD133 and lead to an EMT-like program	(44)
MALAT1	2015	↑N-cadherin	Downregulation of MALAT1 through the activation of PI3K-AKT pathways later results in EMT	(45)
MALAT1	2018	↑Vimentin, MMP9	The upregulation of EMT-related protein(MMP-9 and vimentin) is associated with NF-κB, which would be inhibited after decreasing the expression of MALAT1	(27)
MALAT1	2017	↓E-cadherin, ↑Vimentin, N-cadherin	MALAT1 may promote cell metastasis and result in EMT phenotype via the miR-204/ZEB2 axis	(46)
MALAT1	2016	↓E-cadherin, ↑Vimentin, MMP9	MALAT1 acts as ceRNA of Cdc42 by binding to miR-1 and then lead to EMT.	(47)
HOTAIR	2018	↓E-cadherin,↑Vimentin, β-catenin	TGF-β1 secreted by CAFs, activates TGF-β1/SMAD pathway leading to the positively-regulation of HOTAIR transcription and histone modification of CDK5 signaling pathway.	(28)
HOTAIR	2013	↑Vimentin, fibronectin	HOTAIR upregulated by TGF-β1 acts as a key regulator that controls the multiple signaling mechanisms involved in EMT.	(48)
NEAT1	2017	↓E-cadherin, ↑Vimentin, N-cadherin	IncRNA NEAT1 can induce EMT through the miR-211/HMGA2 axis.	(49)
NEAT1	2017	↓E-cadherin, ↑Vimentin, Fibronectin	FOXN3-NEAT1-SIN3A complex promotes EMT by inhibiting the transcription of downstream target genes GATA3 and TJP1.	(29)
NEAT1	2016	↓E-cadherin, ↑N-cadherin	Increased expression of NEAT1 stimulated EMT and the underlying mechanism is not clear.	(18)
linc-ROR	2017	↓E-cadherin	Downregulation of IncRNA-ROR can inhibit EMT by increasing the expression of a negative regulator miR-205-5p and reducing the expression of ZEB1 and ZEB2 which both capable of binding to E-boxes in the E-cadherin promoter	(50)
linc-ROR	2014	↑E-cadherin, ↑Vimentin, Fibronectin, N- cadherin, aSMA	Linc-ROR overexpression prevents the degradation of mir-205 target genes, including the EMT inducer ZEB2.	(30)
UCA1	2016	↓E-cadherin, ↑ N-cadherin, Vimentin, Snail	UCA1-induced EMT at least partly via activating the Wnt/β-catenin signaling pathway	(51)
UCA1	2018	↓E-cadherin, ↑N-cadherin, Fibronectin	AC026904.1 and UCA1 cooperatively upregulate Slug expression at both transcriptional and post-transcriptional levels.	(31)
TINCR	2019	↓E-cadherin, β-catenin, ↑Vimentin, N- cadherin	TINCR induced by acetylation of H3K27 up-regulates miR-125b, and promotes the EMT process by increasing the expression of Snail-1.	(32)
ANCR	2016	↓E-cadherin, ↑Vimentin	Through influencing the stability of EZH2, ANCR negatively regulated the process of EMT.	(52)
ANCR	2017	↓E-cadherin, ↑N-cadherin, Vimentin	ANCR, as a new downstream molecule of TGF-β, plays an important role in TGF- β1-induced EMT by reducing the expression of RUNX2.	(21)
lnc015192	2018	↓E-cadherin, ↑N-cadherin, Vimentin	lnc015192 could be used as the ceRNA of miR-34a to modulate Adam12, but the detailed mechanism of EMT was not defined.	(36)
LINC01638	2018	↓E-cadherin, ↑Vimentin	LINC01638 interacts with c-Myc to inhibit SPOP-mediated c-Myc degradation and ubiquitination. C-Myc promotes MTDH expression and therefore stimulates Twist1 expression to result in an EMT program.	(37)
lncRNA-HIT	2015	↓E-cadherin, ↑Vimentin	LncRNA HIT can be induced by TGF-β and play a critical role in TGF-β-induced EMT	(53)
NKILA	2018	↓E-cadherin, ↑Vimentin	TGF-β enhances the expression of NKILA, thus inhibiting the overactivation of NF- κB and TGF-β-induced EMT	(39)
ARNILA	2018	↓E-cadherin, ↑N-cadherin	ARNILA promoted EMT by competitively binding to miR-204, leading to the upregulation of Sox4.	(40)
XIST	2018	↓E-cadherin, ↑Vimentin	Decreased expression of XIST stimulated EMT and the underlying mechanism is not clear.	(42)

## LncRNAs in the Distant Metastasis of Breast Cancer

### LncRNAs Implicated in the Lung Metastasis of BC

#### MALAT1

Metastasis-associated lung adenocarcinoma transcript 1 (MALAT1) was first found in non-small cell lung cancer (NSCLC) patients. Functioning as a poor prognosticator, its overexpression could be predictive of a higher stake related to distant metastasis in a series of cancers such as lung cancer, osteosarcoma, colorectal cancer, and especially in non-small cell lung cancer patients (54, 55). Zhang et al. have concluded that MALAT1 is actively involved in multiple physiological processes, such as myogenesis, alternative splicing, synapse formation, and epigenetic modification of gene expression, as well as in multiple pathological conditions (56).

In the past few decades, many *in vitro* and xenograft researches have reported the underlying functions of MALAT1 in regulating invasion, migration, EMT and the metastasis procedure of a diversity of malignancies, and have indicated inconsistent functions of MALAT1 with respect to the growth and progression of tumor cells (57–59). Experiments both *in vitro* and *in vivo* have revealed that MALAT1 is a proliferation promoter, as well as accelerating tumor development and metastasis in triple-negative breast cancer (TNBC). Moreover, it is even negatively correlated with the prognosis of breast cancer patients with an HER-2 positive ER-negative subtype or triple-negative subtype (60). Another study has demonstrated that 17b-Estradiol (E2) with a high level of concentration may inhibit cell growth, invasion and metastasis; in the meantime, the level of MALAT1 is reduced as well either in MCF7 cell lines (Luminal A) or in MDA-MB-231 cell lines (TNBC). Similar effects could be achieved through downregulated MALAT1, so E2 may influence tumor cells through regulating the lncRNA MALAT1 (61).

Xu et al. found that MALAT1 was downregulated in breast tumor cell lines and cancer tissue, and downregulation of MALAT1 in breast cancer cell lines through the activation of phosphatidylinositide-3 kinase-AKT (PI3K-AKT) pathways later results in EMT (58). Recently, MALAT1 was also considered a proinflammatory factor which regulated the lipopolysaccharide (LPS)-induced inflammatory response (62) and EMT process of breast cancer cells (63, 64). The upregulation of EMT-related protein (MMP-9 and vimentin) is associated with NF-κB, which would be inhibited after decreasing the expression of MALAT1 (27). CD133 (PROMININ1), which is one of the general cancer stem cell (CSC) markers, has been reported to possess the ability to facilitate EMT in breast cancer and other malignant tumors (65), and Latorre et al. recently showed that the failure to form or stabilize a repressive complex consisted of MALAT1 and the RNA-binding protein HuR in breast cancer upregulates CD133 and leads to an EMT-like program with the increase of N-cadherin (44).

Some studies showed that MALAT1 inhibited the expression of E-cadherin and induced the expression of vimentin at mRNA and protein levels, while miR-1 inhibited the expression of vimentin and MMP-9 while enhancing the expression of E-cadherin in Western blot results, which can be summarized as MALAT1 and miR-1 having opposite effects on the migration and invasion of breast cancer cells. In conclusion, MALAT1 acts as ceRNA of Cdc42 by binding to miR-1 and then leads to EMT in human breast cancer cell lines (47). miR-204 expression was downregulated by MALAT1 via acting as an endogenous sponge. MiR-204 inhibited the expression of ZEB2 by binding to the non-coding region of ZEB2 3-UTR. Therefore, MALAT1 regulated the miR-204/ZEB2 axis in breast cancer. In view of the fact that ZEB2 is a key factor in EMT, it was speculated that MALAT1 may promote cell metastasis and result in an EMT phenotype via the miR-204/ZEB2 axis (46).

As part of *in vivo* studies, Zhang et al. generated mice models with a deleted-3-kb genomic site encompassing the 5′end of lncRNA MALAT1 as well as its promoter region (66). Another study reported that the systemic knockdown or genetic loss of MALAT1 in the MMTV (mouse mammary tumor virus)-PyMT (polyomavirus middle T antigen) (67) rats model led to slower growth of tumor cells as well as a reduction in lung metastases (68). They found a diminishment of branching morphogenesis in Her2/neu-amplified and MMTV-PyMT tumor tissues and cell migration accompanied by an enhancement of cell adhesion in MALAT1-loss cells; however, the potential mechanism regarding the reduced metastasis remained unclear. It is considered that this MALAT1-loss model is usually accompanied by the upregulation of substantial adjacent genes of MALAT1, including Neat1, Tigd3, Frmd8, Ehbp1l1, and so on (66). In order to explore whether this high level of expression was caused by the deletion of MALAT1 or the loss of regulatory sequences for its adjacent genes, Kim et al. adopted a MALAT1-knockout animal model wherein a transcriptional terminator was inserted 69-bp downstream of the transcriptional start region of MALAT1. When the expression of MALAT1 was restored in breast tumor tissue, the distant metastasis in the lung was reduced. Besides, they proposed that MALAT1 suppresses metastasis in a TEAD-dependent manner, which associates and inhibits the prometastatic transcription factor TEAD through binding to its target gene promoters and coactivator YAP (26).

The opposite observation results from disparate studies suggested the complicacy of MALAT1 in BC, which may be according to the specific tumor subtypes or different cell types.

#### HOTAIR

HOTAIR, which is an lncRNA in the mammalian HOXC locus that targets and binds to polycomb repressive complex 2 (PRC2) to the HOXD locus, is located on a disparate chromosome (69).

HOTAIR expression is upregulated in breast cancer tissue and its metastasis, and could also predict the eventual metastasis and death according to different expression levels. LncRNA HOTAIR has been proven through interacting with PRC2, which can promote breast cancer progression (20). For example, the interaction of HOTAIR with PRC2 or LSD1 can be inhibited by HOTAIR targeting small molecular inhibitors, thereby reducing the metastasis of breast cancer (70).

Some findings suggest that HOTAIR is involved in the activation of the genetic process that promotes EMT. Padua Alves et al. have shown that HOTAIR is an important regulator of EMT-related genes in breast cancer cells, and transforming growth factor-β1 (TGF-β1) supports this role of HOTAIR (48). TGF-β-1 induced the upregulated expression of HOTAIR, which may be a central event in promoting PRC2 to inhibit E-cadherin gene expression. This hypothesis is supported by other extended analyses of gene expression profiles, PRC2 occupation, and H3K27me3 (20). In these analyses, they observed the activation of EMT in cells overexpressing HOTAIR. Other experiments generally support the view that TGF-β1 secreted by cancer-associated fibroblasts (CAFs) activates the TGF-β1/SMAD pathway in breast cancer cells, whereby leading to the positive regulation of HOTAIR transcription and histone modification of the CDK5 signaling pathway. Therefore, EMT can be induced to promote the progression and lung metastasis of breast cancer *in vivo* (28). Moreover, HOTAIR can inhibit the expression of miR-7. In turn, can significantly induce the expression of E-cadherin, and reverse EMT by downregulating the STAT3 pathway (43).

#### NEAT1

Nuclear-enriched abundant transcript 1 (NEAT1) is a sub-nuclear structure localized exclusively to paraspeckles (71). Its function is that of modulating RNA splicing as well as transcription. It has been proposed that NEAT1 functions as an oncogenic lncRNA in various kinds of malignancies, such as BC, and it is suggested to induce EMT in cancer progression (72).

Several studies have reported that lncRNA NEAT1 accelerates the proliferation and progression of malignancies. EMT is one of the essential contributors to invasion and metastasis in breast carcinoma (25). Zhang et al. found that the expression of EMT markers was altered according to the expression of lncRNA NEAT1. The expression of E-cadherin was increased and N-cadherin was decreased by inhibiting lncRNA NEAT1, suggesting that lncRNA NEAT1 might regulate EMT in BC; however, the underlying mechanism remains unclear (18).

HMGA2 has been reported to regulate EMT transcription factor (EMT-TF) networks such as Snail1, Snail2, ZEB1, ZEB2, and Twist1 (58). There was mutual inhibition between NEAT1 and miR-211. In addition, EMT inducer HMGA2 was identified as a downstream target of miR-211. This concluded that lncRNA NEAT1 can induce EMT through the miR-211/HMGA2 axis (21). As part of an *in vivo* experiment, cells transduced with sh-NEAT1 were inoculated into animal models through the tail vein, and it was found that compared with the control group, the experimental group generated smaller distant metastatic colonies in the lung, meaning that inhibiting the activity of lncRNA NEAT1 reduced the metastatic ability of cancer cells *in vivo* (21).

FOXN3 is a transcription inhibitor associated with SIN3A repressor complexes in estrogen receptor-positive (ER+) cells. It has been shown that an FOXN3–NEAT1–SIN3A complex promotes the invasion of breast cancer cells and EMT *in vitro* and the proliferation and metastasis of breast cancer *in vivo* by inhibiting the transcription of downstream target genes GATA3 and TJP1. After inoculating the engineered MCF-7 breast cancer cells into the left abdominal mammary fat pad, it was found that overexpression of either NEAT1 or FOXN3 efficiently promoted lung metastasis in animal models (29).

#### Linc-ROR

It has been reported for lncRNA-regulator of reprogramming (lincRNA-ROR) that the expression is upregulated in several different solid tumors, which suppresses the invasion of a tumor through reducing the EMT marker's expression (73). Additionally, lincRNA-ROR also potently promotes the invasion and distant metastasis ability of BC through the EMT program.

Hou et al. first identified the role of linc-ROR in the control of EMT and metastasis in breast cancer cells. Linc-ROR is associated with miRNPs and acts as an endogenous RNA competing with mi-205. Specifically, linc-ROR overexpression prevents the degradation of mir-205 target genes in breast cancer cells, including the EMT inducer ZEB2. Therefore, linc-ROR is an important regulator of EMT and promotes the progression and metastasis of breast cancer by regulating miRNAs (30). It has been verified that in MDA-MB-231 cells, the downregulated expression of lncRNA-ROR can inhibit the EMT of breast cancer by increasing the expression of negative regulator miR-205-5p and reducing the expression of ZEB1 and ZEB2, key EMT-TFs that, more than others, play a central role in controlling EMT activation (74), both of which include two zinc finger domains capable of binding to E-boxes in the gene promoter region, such as the E-cadherin promoter (50).

#### UCA1

Urothelial carcinoma-associated 1 (UCA1), which is an lncRNA that contains three exons and encodes two transcripts (75), was first detected overexpression in bladder cancer (76). Its expression level is related to cellular proliferation and migration (77). The expression levels of AC026904.1 and UCA1 in metastatic breast cancer were reported to be higher than those in non-metastatic breast cancer (31).

Huang et al. proved UCA1 to be an oncogenic lncRNA in BC either *in vitro* or *in vivo*. UCA1 bound to hnRNP I (heterogeneous nuclear ribonucleoprotein I) and formed a complex which made itself more stabilized. While promoting the translational ability of p27 through hnRNP I alone, the protein content of p27 was widely suppressed when it interacted with UCA1 (78). Current research has observed that TGF-β activates UCA1 and AC026904.1 through Smad and ERK pathways, respectively. AC026904.1 activates the cis-transcription of the Slug gene in the form of eRNA. UCA1 promotes the expression of Slug in breast cancer by directly titrating miR-1 and miR-203a at the post-transcriptional level. Both of them cooperated with each other to upregulate the expression of Slug at the transcriptional and post-transcriptional levels (31). The Wnt/ β-catenin signaling pathway is recognized as a common signaling pathway related to EMT and the metastasis of breast cancer (79, 80). After transfection of si-UCA1 into MDA-MB-231 cells, the expression of negative regulators p-gsk-3 β and gsk-3 β in the Wnt signaling pathway was significantly increased. At the same time, UCAl gene knockout inhibited the protein expression of β-catenin and its downstream genes (including cyclinD1 and MMP7) (51).

#### TINCR

TINCR (terminal differentiation-induced non-coding RNA) is a spliced lncRNA which produces a 3.7-kb transcript. It was initially discovered from well-differentiated human somatic tissue and was proven to be an indispensable component of normal differentiation of the epidermis (81). Liu and his colleagues reported TINCR to be an oncogenic factor for BC (82); however, whether it is because of drug resistance or resistance-induced tumor metastasis remains unknown.

Compared with other sensitive cells, TINCR was critically upregulated in trastuzumab-resistant tumor cells. While the drug resistance and EMT program could be reversed through the knockdown of TINCR, Dong et al. demonstrated that TINCR induced by acetylation of H3K27 upregulates miR-125b, and promotes the EMT process by increasing the expression of Snail-1. Experimental distant metastasis in the lung was formed by injections into the lateral tail vein of mouse models. *In vivo*, compared to the sh-NC group, both the deep lung metastasis and the visible metastasis on the surface of the lung were distinctly lesser in the experimental group (32).

#### BORG

BMP/OP-responsive gene (BORG) in a C2C12 mouse myoblast cell line which trans-differentiates into osteoblastic cells reacts to bone morphogenetic proteins (BMPs) (83), particularly alters the proliferation of disseminated BC cells.

Alex et al. revealed that high expression of BORG is closely associated with recurrent and metastatic disease, making it a potential biomarker which is able to identify women who are at high risk of developing BC with high invasiveness or metastatic ability. In theory, BORG induces the metastatic colonies of potent BC cells by activating the transcriptional repressive activity and localization of TRIM28, which combines with BORG and leads to a great amount of changes in cancer progression. In addition, downregulated BORG in metastatic BC enhances their metastatic ability in animal models, indicating that BORG could promote alterations at both epigenetic and genetic levels, therefore underlying the development of recurrent and metastatic BC (33).

#### LincIN

LincIN is a long intergenic non-coding RNA between ITGB1 and NRP1 (LincIN), using a high-density SNP array-based gene expression approach to assess the lncRNA transcriptome in paired tumor tissue vs. normal samples.

Compared to adjacent normal tissue, it is frequently observed with highly expressed LincIN in tumors, and it is reported that LincIN is significantly correlated with aggressive BC. Moreover, analysis of TCGA data also supports that upregulation of LincIN is associated with poor prognosis in BC patients. Conversely, deletion of LincIN suppresses the invasion and migration of tumor cells in experimental studies, and transcriptome analysis of the LincIN-deletion tumor cells agrees with this result. Importantly, the loss of LincIN decreases the amount of lung metastasis in tail vein injected- mice models. A study suggested that LincIN exerts a critical role in translational alterations by cooperating with NF90 to suppress the translation of p21, and the upregulation of nuclear p21 by LincIN knockdown may be related to a less aggressive phenotype in a metastasis model (34).

#### ANCR

An lncRNA termed anti-differentiation ncRNA (ANCR or DANCR), an 855-nucleotide lncRNA, was first discovered during the differentiation process with its downregulated expression (84).

Li and his colleagues revealed that ANCR exerts a pivotal role in BC progression and metastasis, mostly through diminishing EZH2 (enhancer of zeste homolog 2) stability. EZH2, as a pivotal inducer and regulator of EMT at the epigenetic level, contributes to multiple cancer metastases. They initially found that in contrast to normal breast tissue, the expression level of ANCR was lower both in BC cell lines and in BC samples. ANCR-mediated EZH2 degradation may play a key role in weakening the ability of initiating EMT and metastasis programs in breast cancer cells. ANCR interacts with EZH2 to promote the binding of CDK1 to EZH2 and its phosphorylation at Thr-345 and Thr-487 sites, which leads to the degradation of EZH2. Through influencing the stability of EZH2, ANCR negatively regulated the process of EMT (35).

Another study showed that ANCR, as a new downstream molecule of TGF-β, plays an important role in TGF-β1-induced EMT by reducing the expression of RUNX2. TGF-β1 induces the increasing expression of HDAC3; HDAC3 then binds to the promoter region of ANCR which inhibited the transcription of ANCR. Besides, ANCR attenuates the TGF-βsignaling pathway at least in part by inhibiting the phosphorylation of Smad2/3. In an *in vivo* study, the expression level of RUNX2 in lung tissue sections was measured, uncovering a decreasing level of RUNX2 in the lung tissue of mice models which had been injected with MDA-MB-231-ANCR cells (52).

#### Lnc015192

In a recent study, Huang et al. found that Adam12 is overexpressed in breast cancer cell lines, and promotes mesenchymal-epithelial transition (MET) and inhibits migration and invasiveness. The knockout of Adam12 and lnc015192 inhibits migration, invasion and EMT in breast cancer cells. It has been shown that lnc015192 could be used as the ceRNA of miR-34a to modulate Adam12, but the detailed mechanism of EMT has not been defined. In order to explore the influence of lnc015192 and Adam12 on tumor metastasis *in vivo*, either lnc015192 or Adam12-deletion 4T1 cells were injected into mice models, and the results showed that a smaller number of lung metastatic focuses were induced in sh-lnc015192-injected or sh-Adam12-injected animal models than in the control group, suggesting that the loss of lnc015192 or Adam12 suppresses the invasion, migration and metastasis ability of BC cells (36).

#### LINC01638

A validated lncRNA, LINC01638 (long intergenic non-protein coding RNA 1683), was reported with low expression in various normal human tissues (85) and may be associated with the EMT program of HCC cells (86).

Current research has suggested that LINC01638 is expressed at a high level in TNBC cells and tissue. LINC01638 maintains the mesenchymal characteristics of TNBC cells, containing a cancer stem cell-like state and enriched EMT signature. The knockdown of LINC01638 inhibits tumor cell proliferation and invasiveness either *in vivo* or *in vitro*. Overexpression of LINC01638 is regarded as a predictor of poor prognosis of BC patients. In theory, LINC01638 interacts with c-Myc to inhibit SPOP-mediated c-Myc degradation and ubiquitination. C-Myc promotes the expression of MTDH (metadherin) at a transcriptional level and, therefore, stimulates Twist1 expression, resulting in an EMT program.

In addition, to assess the impact of LINC01638 on BC metastasis *in vivo*, LINC01638-deletion MDA-MB-231 cells were inoculated into animal models via the tail vein. A smaller number of lung metastatic nodules were revealed from the experimental group than from the control group after several days, but restoring the expression of c-Myc diminished the impact of LINC01638 deletion on tumor metastasis *in vivo*.

These findings verified LINC01638-mediated signal transduction and emphasized the key role that LINC01638 played in TNBC development and metastasis (37).

#### LncRNA-HIT

In addition, lncRNA-HIT (HOXA-associated transcript induced by TGF-β) also plays a key role in TGFβ-induced EMT. E-cadherin was identified as being one of the major targets of lncRNA-HIT. The effects of lncRNA-HIT on EMT, invasion and migration in a 4T1 orthotopic mouse xenograft model were rescued by the introduction of ectopic E-cadherin. These results suggest that lncRNA-HIT can be induced by TGF-β and play a critical role in TGF-β-induced EMT (53).

### LncRNAs Implicated in the Liver Metastasis of BC

#### NKILA

Nuclear factor-κB interacting lncRNA (NKILA) is a main suppressing checkpoint for NF-κB activation in BC. A gene at chromosome 20q13 encoded NKILA, which first exerted the antimetastatic abilities of breast cancer cells. NKILA is a novel lncRNA which was detected recently by Liu et al. (38), who attempted and successfully proposed that lncRNA NKILA suppresses the activation of NF-κB signaling through combining with the NF-κB/IκB complex to cover up the phosphorylation regions of IκB; therefore, the NF-κB/IκB complex is stabilized. Additionally, both tumor samples and *in vivo* studies validated that the downregulation of NKILA in high-grade BC promotes distant metastasis (38).

A recent study found that NF-κB plays an important role in TGF-β-induced EMT. TGF-β enhanced the expression of NKILA, thus inhibiting the overactivation of NF-κB by interacting with I-κB. The expression of NKILA controls the switch “on and off,” negatively regulating NF-κB. Additionally, the upregulation of NKILA expression notably decreased liver metastasis induced by TGF-β of a malignant tumor *in vivo*. Similar to the outcomes of animal models, the expression of NKILA was negatively associated with different EMT phenotypes in BC tissue. In conclusion, it has been shown that lncRNA NKILA can modulate the TGF-β-induced EMT of breast cancer (39).

#### ARNILA

Androgen receptor negatively induced lncRNA (ARNILA) is located in the cytoplasm of TNBC tissues and cells, which have been demonstrated to promote invasion, EMT and metastasis in *in vivo* and *in vitro* experiments. In addition, ARNILA was reported to function as a ceRNA for miR-204 to promote its target gene Sox4's expression, which subsequently contributed to the EMT program and induced the progression of breast cancer, thereby facilitating the invasion, EMT and metastasis of TNBC (40).

#### MALAT1

In a recent study, which identified MALAT1 as a target of miR-1, it was found that the expression level of miR-1 was negatively correlated with the expression of MALAT1 in BC samples. MiR-1 suppressed the development of BC, decreased cell motility and activated apoptosis by regulating MALAT1. The knockdown of MALAT1 could partly imitate the tumor-inhibitive impact of miR-1. As part of *in vivo* metastatic experiments, MCF7 cells transduced stably with miR-1 formed fewer and smaller lung and liver metastases than the control group. Such outcomes indicated that miR-1 could suppress the metastasis of BC cells by regulating MALAT1 *in vivo* (19).

### lncRNAs Implicated in the Brain Metastasis of BC

#### lnc-BM

LncRNA associated with brain metastasis (Lnc-BM) has been demonstrated to be a prognostic factor of the progression of brain metastasis in BC patients. In preclinical experiments, upregulated expression of Lnc-BM promoted BCBM, while the knockdown of Lnc-BM with nanoparticle-encapsulated siRNAs effectively improved BCBM in murine models. In BC cells, Lnc-BM drove the STAT3-dependent expression of CCL2 and ICAM1, which acts as a mediator in the process of recruitment of macrophages and vascular co-option in the cerebrum. Macrophages which were recruited inversely produced IL-6 and oncostatin M, thereby further activating the Lnc-BM/JAK2/STAT3 pathway and promoting BCBM by mediating interaction between BC cells and the brain microenvironment (41).

#### XIST

X-inactive-specific transcript (XIST) is a lncRNA that participates at the beginning of X chromosome inactivation during early embryogenesis. Numerous human malignant tumors are accompanied by the deficiency of inactivated X chromosomes (Xi) as well as the duplication of active X chromosomes (87). Such a phenomenon is more common in several cancers such as breast cancer.

Here, Xing et al. found that XIST was notably decreased in BM tissue from patients with BC. The expression level of XIST was inversely associated with BM in BC patients. A loss of XIST preferentially facilitated the BM growth of XIST-high cells in xenograft animal models. Additionally, deletion of XIST in the breast of mice models enhanced the growth of BC and BM. Downregulation of XIST activated EMT and stimulated c-Met through stabilizing MSN-mediated protein, which contributed to the activation of tumor cells' stemness. Deletion of XIST also drove the secretion of exosomal miRNA-503, which consequently triggered M1–M2 polarization of microglia. However, the underlying mechanism of EMT remains unclear (42).

#### Others

In addition, there are some other lncRNAs which have been reported to be associated with the brain metastasis of breast cancer patients, such as NCT02915744 III, which is related to metastatic BC with brain metastases, and NCT02000882 II, which is found in TNBC patients with brain metastases (88).

### LncRNAs Implicated in Other Metastasis of BC

#### HOTAIR

MiR-7, which was initially detected from human MDA-MB-231 and MCF-7 cell lines, was lowly expressed in breast cancer stem cells (BCSCs). Zhang and his colleagues found that MiR-7 suppressed the metastasis of BCSCs in many different organs, such as kidneys, lungs as well as adrenal glands, in NOD/SCID mice. Moreover, downregulated miR-7 may be indirectly owing to HOTAIR through regulating the expression level of HoxD10, which enhances miR-7 expression in BCSCs (43).

## Conclusion

In the past few decades, lncRNAs have experienced a long history, from being regarded as non-functional (6) to being confirmed as an indispensable contributor to the development and progression of a variety of malignant tumors, attracting a considerable amount of attention from multiple investigators who have devoted themselves to research on lncRNAs and malignant tumors, especially tumor metastasis (89), which would directly result in cancer-related death.

In this review, we briefly illustrated those lncRNAs involved in regulating the metastatic ability of breast cancer and tried to describe the potential mechanisms, especially through the EMT process, as well as attempting to explore some novel strategies for targeting highly invasive BC. Depending on the screening results of lncRNAs, a large number of them are demonstrated to function as a promoter in the process of invasion, migration, EMT or metastasis of BC, while a small number have contradictory effects to inhibit the metastasis of BC. They regulated the distant metastatic process at different levels such as transcription levels or post-transcription levels, and it seems that there are several signaling pathways and transcription factors that are similar among different lncRNA-involved pathways, which may be novel therapeutic targets for cancer patients in the future. However, the underlying mechanisms regarding how most of the lncRNAs regulate breast cancer remain under-defined (90).

Some studies verified whether different lncRNAs affect the distant metastasis of breast cancer through injecting the breast cancer cells with or without lncRNAs into the animal models, and observe the size and the quantity of the distant metastatic colonizations to discover the underlying mechanisms among the process of distant metastasis (28, 35, 37, 46). It has been reported that EMT mediates the dissemination of tumor cells from primary lesions, while MET is essential for the seeding of circulating tumor cells to distal organs (91–93). We intended to summarize the metastatic mechanisms to different organs accordingly, however, most of them focused on the initiation mechanism of metastasis, such as EMT, there are less studies tried to find the mechanism associated with the different metastatic organs. Huang et al. reported that lnc015192 knockdown enhanced the MET process in the lung metastasis while the underlying mechanism remains unclear (36). Another study showed that ANCR, as a new downstream molecule of TGF-β, plays an important role in TGF- β1-induced EMT by reducing the expression of RUNX2. They uncovered a reduced RUNX2 level in nude mice lung tissues injected with MDA-MB-231-ANCR cells (52). Brain metastasis has been reported with several organ-specific lncRNAs in our review, XIST expression was found significantly downregulated in brain metastatic lesions compared with other metastatic tumors based on an organ-specific cohort analysis, deletion of XIST also drove the secretion of exosomal miRNA-503, which consequently triggered M1–M2 polarization of microglia in the brain (42). Lnc-BM drove STAT3-dependent expression of CCL2and ICAM1, acting as a mediator in the process of recruitment of macrophages and vascular co-option in the cerebrum. And then promoted BCBM via activating the Lnc-BM/JAK2/STAT3 pathway (41). It seems that some lncRNAs are still related with organ-specific metastasis in breast cancer patients, but without enough studies to support an exact classification, further studies should pay more attention on this direction.

The aforementioned studies demonstrated that the lncRNAs upregulated or downregulated in BC cells or tissue are promising targets for therapy and predictors of prognosis. Nevertheless, the current problem with their clinical application is how to find a way in which to detect these lncRNAs conveniently. As we all know, lncRNAs that are usually located in the cytoplasm or nucleus of tumor cells may interact with DNA, mRNA, miRNA or protein to perform their various functions (94, 95). Nowadays, several scientists are exploring the detection of lncRNAs in the circulatory system. LncRNA HOTAIR has been detected in patients' serum with various malignant tumors and might be a potential biomarker for diagnosis and prognosis prediction of BC (96). Extracellular lncRNA-SNHG14 was capable of being equipped in exosomes and then transmitted to sensitive cells, consequently diminishing drug resistance (97). In addition, circulating PVT1 DNA increases notably in the serum of BC patients to be detected. Compared with PVT1 RNA, DNA is a more common form of the PVT1-derived segment (49). These findings totally indicate that lncRNAs-relevant factors play a critical role in BC and are possible targets for examination and therapy, which might be a more convenient method to act as a prognosticator for BC patients in the future.

## Author Contributions

All authors participated in designing the concept of this manuscript. YW, LW, CY, and KH reviewed the literature and drafted the article. CP, SZ, and AS finalized the paper and provided suggestions to improve it.

### Conflict of Interest Statement

The authors declare that the research was conducted in the absence of any commercial or financial relationships that could be construed as a potential conflict of interest.
